# Advanced Symptom Management System for Patients with Malignant Pleural Mesothelioma (ASyMSmeso): Mixed Methods Study

**DOI:** 10.2196/19180

**Published:** 2020-11-12

**Authors:** Roma Maguire, John Connaghan, Anne Arber, Naomi Klepacz, Kevin G Blyth, John McPhelim, Paul Murray, Hitasha Rupani, Anoop Chauhan, Peter Williams, Laura McNaughton, Kirstie Woods, Anne Moylan

**Affiliations:** 1 Department of Computing and Information Sciences University of Strathclyde Glasgow United Kingdom; 2 Faculty of Health and Medical Sciences University of Surrey Guildford United Kingdom; 3 Glasgow Pleural Disease Unit Queen Elizabeth University Hospital NHS Greater Glasgow and Clyde Glasgow United Kingdom; 4 Institution of Cancer Sciences University of Glasgow Glasgow United Kingdom; 5 University Hospital Hairmyres NHS Lanarkshire East Kilbride United Kingdom; 6 Ashford and St Peter's NHS Trust Surrey United Kingdom; 7 Portsmouth Hospitals University NHS Trust Portsmouth United Kingdom; 8 Faculty of Engineering and Physical Sciences University of Surrey Guildford United Kingdom; 9 University Hospital Monklands NHS Lanarkshire Airdrie United Kingdom

**Keywords:** malignant pleural mesothelioma, patient reported outcome measures, cancer, mobile health, telemedicine, symptom monitoring

## Abstract

**Background:**

Patients with malignant pleural mesothelioma (MPM) have a life-limiting illness and short prognosis and experience many debilitating symptoms from early in the illness. Innovations such as remote symptom monitoring are needed to enable patients to maintain wellbeing and manage symptoms in a proactive and timely manner. The Advanced Symptom Management System (ASyMS) has been successfully used to monitor symptoms associated with cancer.

**Objective:**

This study aimed to determine the feasibility and acceptability of using an ASyMS adapted for use by patients with MPM, called ASyMSmeso, enabling the remote monitoring of symptoms using a smartphone.

**Methods:**

This was a convergent mixed methods study using patient-reported outcome measures (PROMs) at key time points over a period of 2-3 months with 18 patients. The Sheffield Profile for Assessment and Referral for Care (SPARC), Technology Acceptance Model (TAM) measure for eHealth, and Lung Cancer Symptom Scale-Mesothelioma (LCSS-Meso) were the PROMs used in the study. Patients were also asked to complete a daily symptom questionnaire on a smartphone throughout the study. At the end of the study, semistructured interviews with 11 health professionals, 8 patients, and 3 carers were conducted to collect their experience with using ASyMSmeso.

**Results:**

Eighteen patients with MPM agreed to participate in the study (33.3% response rate). The completion rates of study PROMs were high (97.2%-100%), and completion rates of the daily symptom questionnaire were also high, at 88.5%. There were no significant changes in quality of life, as measured by LCSS-Meso. There were statistically significant improvements in the SPARC psychological need domain (*P*=.049) and in the “Usefulness” domain of the TAM (*P*=.022). End-of-study interviews identified that both patients and clinicians found the system quick and easy to use. For patients, in particular, the system provided reassurance about symptom experience and the feeling of being listened to. The clinicians largely viewed the system as feasible and acceptable, and areas that were mentioned included the early management of symptoms and connectivity between patients and clinicians, leading to enhanced communication.

**Conclusions:**

This study demonstrates that remote monitoring and management of symptoms of people with MPM using a mobile phone are feasible and acceptable. The evidence supports future trials using remote symptom monitoring to support patients with MPM at home.

## Introduction

Malignant pleural mesothelioma (MPM) is an asbestos-related cancer that affects the pleura surrounding the lung. Approximately 2700 people are diagnosed with mesothelioma each year in the United Kingdom; of those, 89% have MPM, most of whom are men [1,2]. MPM may progress in different ways; some people may have a period of stability, while others have a more progressive illness with a limited prognosis [3]. Many patients experience several symptoms simultaneously in addition to a high burden of emotional and psychological distress from early in the illness [4-6]. The most commonly reported symptom of MPM is chest pain, followed by breathlessness, fatigue, weight loss, and cough; these are accompanied by a reduction in quality of life resulting in considerable need for support [7-11]. Early supportive care interventions are essential to those with MPM so that a good quality of life is maintained for as long as possible [12]. With a shift in care from hospitals to local community settings, many patients with MPM must engage in self-care activities at home to prevent or reduce the severity of symptoms and treatment side effects and must make important decisions such as when to contact health services. Use of technological devices such as smartphones and tablets enables the monitoring of symptoms and increases the capacity for self-care as well as enhancing communication with health professionals.

Remote symptom monitoring is now available to support patients, such as those with MPM, throughout their illness with the use of devices such as smartphones to remotely collect and send data to health care providers for diagnostic interpretation or monitoring purposes. Remote symptom monitoring has been proven to be effective in the management of people with cancer being monitored at home [13]. A study by Basch et al [13] found that people with advanced cancer who reported their symptoms to health professionals using tablets were less likely to visit accident and emergency or be hospitalized, remained on chemotherapy for longer, and had improved survival. Furthermore, the benefits of using remote symptom monitoring have been demonstrated in large reviews of people with cancer and a range of life-limiting illnesses [14,15].

A remote symptom monitoring system called the Advanced Symptom Management System (ASyMS) has been developed by the authors. A study of the use of remote symptom monitoring of patients with lung cancer found that ASyMS led to improved symptom management and enhanced communication with health professionals [16]. ASyMS is one of the most evolved and tested remote symptom monitoring systems in the field [17-19]. In this article, we discuss a study where we adapted ASyMS for patients with MPM, called ASyMSmeso, designed to meet the needs for symptom monitoring to support patients with MPM at home.

## Methods

### Study Aim and Objectives

The overall aim of the study was to adapt ASyMS for people with MPM (ASyMSmeso) and determine the feasibility and acceptability of integrating ASyMSmeso into oncology care delivery.

Specific objectives of the study were to (1) explore the experiences and perceptions of people with MPM, their carers, and health professionals while using the ASyMSmeso system through semistructured interviews with people with MPM, their carers, and health professionals; (2) describe changes in patient-reported outcome measures (PROMs; symptoms, supportive care needs, technology acceptance) over time for people with MPM using the ASyMSmeso system (Sheffield Profile for Assessment and Referral for Care [SPARC], Technology Acceptance Model [TAM] measure for eHealth, Lung Cancer Symptom Scale - Mesothelioma [LCSS-Meso]); (3) explore the impact of ASyMSmeso on processes and organization of care and the workforce using semistructured interviews with health professionals; and (4) collect information such as response rates, recruitment rates, adherence to the intervention, compliance to the study using data automatically collected by the system on user interactions, and alerts to inform a future trial of ASyMSmeso.

### Sample

The study aimed to recruit a purposive sample of up to 45 people with MPM from 4 clinical sites across England and Scotland. This sample size was considered acceptable for a feasibility study [20]. The inclusion criteria for the study were that patients had received a definitive diagnosis of MPM, were deemed by a clinician to be both physically and psychologically able to participate in the study, and were predicted to survive for at least 6 months. We recruited 18 patients to the study.

### The ASyMSmeso Intervention

**Figure 1:**
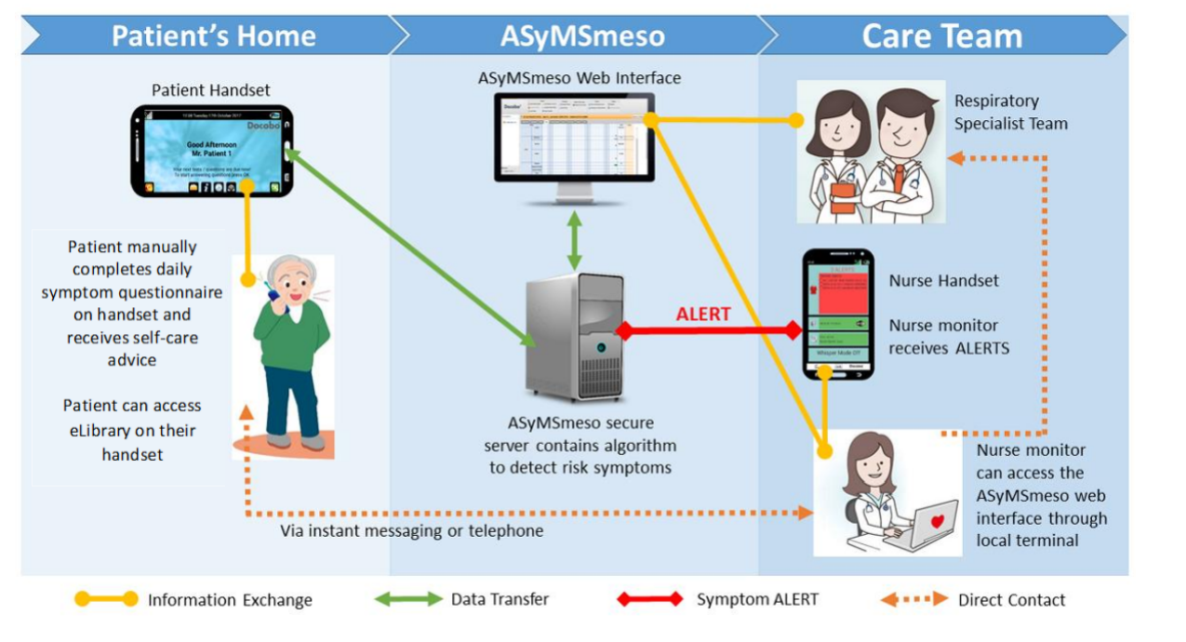
The Advanced Symptom Management System for patients with malignant pleural mesothelioma (ASyMSmeso) intervention process.

The ASyMSmeso intervention is outlined in Figure 1. The patients used their patient handset (Samsung Galaxy J3 mobile phone) to complete a daily symptom questionnaire (DSQ) accessed through an application pre-installed on the mobile phone by the research team. The patients were required to complete a DSQ using their handset every day and at any time they felt unwell. Data from the DSQ were received by the ASyMSmeso secure server and processed by the predefined alerting algorithm. Any symptom or symptom combination meeting the algorithm’s conditions for alerting automatically generated an alert on the dedicated mobile phone handset carried by the responding health professional at the patient’s hospital. The handset played an audio attention prompt on receipt of the alert. The responding health professional then acted on the alert within the pre-agreed 24-hour timeframe. The health professional used a hospital desktop computer to view the patient’s symptom alert report and DSQ responses through a secure web-based clinician’s dashboard before contacting the patient directly via telephone or text message to advise them on how to manage their symptoms. For symptoms that could be self-managed by the patient, the health professional was able to offer self-care advice. The patient could access self-care information at any time via the electronic library (eLibrary) section of the ASyMSmeso application installed on their patient handset and were also able to view graphs of their symptom profile over time. The responding health professional closed the alert by updating the patient record using the clinician dashboard.

### Procedure to Create the Daily Symptom Questionnaire and Alerting Algorithm

The symptoms included in the DSQ (breathlessness, pain, cough, sweating, fatigue, appetite, issues with indwelling pleural catheters, and constipation) and the alerting algorithm were informed by a literature search conducted by the research team and then refined with 3 focus groups with patients, carers, and health professionals (6 patients, 8 carers, 2 health professionals) before finally being reviewed by an expert panel of clinicians (3 respiratory consultants, 2 lung cancer nurse specialists, 2 mesothelioma nurse specialists) who agreed on the final contents of the DSQ and rules for the algorithm. It was agreed that daily monitoring of symptoms was appropriate as it enables early intervention at the start of the symptom trajectory, as demonstrated in our previous studies with ASyMS (16-18). The expert panel also agreed that an appropriate alert response time for a health professional responding to a patient with MPM is 24 hours.

### Ethics

The ASyMSmeso intervention was delivered in addition to and complemented the patients’ standard care. For any symptoms that required immediate medical attention, patients were asked to follow standard guidelines for their local area in the monitoring, management, and reporting of symptoms. Ethical approval was granted by West of Scotland Research Ethics Committee (reference number 17/WS/0077).

### Data Collection

After providing informed consent, participants were asked to complete PROMs at baseline (T1), 6 weeks (T2), and the end of the study (T3). Midway through the study, we submitted an ethical amendment asking to reduce the period that people with MPM used the system, from 3 months to 2 months, to enhance recruitment within the remaining study timelines. For those patients participating in the study for 2 months, data were collected at baseline and the end of study (8 weeks) only. Patients completed the following PROMs: LCSS-Meso, SPARC, and TAM for eHealth.

#### LCSS-Meso

The LCSS-Meso is an 8-item patient scale that evaluates 5 domains, including overall symptomatic distress, functional activities, and global quality of life. Studies indicate it takes between 3 minutes and 8 minutes to complete [21].

#### SPARC 

SPARC is a holistic needs assessment tool that covers physical, psychological, social, spiritual, and financial issues, with estimated completion times of <15 minutes [22,23].

#### TAM for eHealth

The TAM for eHealth questionnaire measures perceptual constructs from the information technology acceptance models: intrinsic motivation, perceived ease of use, perceived usefulness/extrinsic motivation, and behavioral intention to use eHealth. The original scale was revised from 20 to 12 items to keep it short and more practical for real-world situations. The TAM has been adapted for patient groups [24,25].

#### DSQs

In addition to the PROMs, we also analyzed the DSQs, and these formed part of the data analysis.

#### Demographic and Clinical Data

A health professional at the clinical site also completed a clinical and demographic questionnaire capturing patient data concerning age, gender, marital status, number and age of children, education level, occupation, diagnosis, stage of disease, length of time since diagnosis, treatment, Eastern Cooperative Oncology Group (ECOG) performance status, medications, and existing comorbidities (Table 1).

**Table 1:** Demographic characteristics of participants and their Eastern Cooperative Oncology Group (ECOG) status (N=18).

Characteristics			Results, n (%)
**Age (years)**			
	55-64	4 (22)	
	65-74	8 (44)	
	75-84	5 (28)	
	Unknown	1 (6)	
**Gender**			
	Male	13 (72)	
	Female	5 (28)	
**Marital status**			
	Married	15 (83)	
	Widowed	1 (6)	
	Divorced	1 (6)	
	Co-habiting	1 (6)	
**Education**			
	Schooling incomplete	2 (11)	
	Finished high school	5 (28)	
	Further education	2 (11)	
	University	4 (22)	
	Trade qualification	1 (6)	
	Unknown	4 (22)	
**ECOG Status**			
	0 - Fully active	6 (33)	
	1 - Restricted but ambulatory	11 (61)	
	2 - Unable to carry out work activities	1 (6)	

#### Semistructured Interviews and Focus Groups

At the end of the study, patients and/or carers and health professionals were invited to take part in semistructured interviews and focus groups to discuss their experience with the system.

### Study Recruitment

A total of 54 people with MPM across the 4 clinical sites were approached to take part in this study. Of these, 18 patients consented and participated (response rate, 33.3%). Reasons for refusal included not wanting to deal with technology (2/36, 6%) and not wanting a daily reminder of symptoms (2/36, 6%); 32 (32/36, 89%) did not give a reason for not participating. Of the 18 participants, 14 were recruited to the 3-month protocol, and 4 were recruited to the 2-month protocol. Participants were predominantly male (13/18, 72%), with an average age of 71.6 years (see Table 1).

### Data Analysis

Summary statistics are reported for each time point for each outcome in the 3 PROMs used in the study. Changes in PROMs from baseline (T1) to T2 and T3 were assessed using a Wilcoxon matched pairs test. Male participants were compared to female participants for changes in PROMs using a Mann-Whitney U test. The associations between the change in each PROM at T2 and T3 with age, education, and ECOG status was assessed using Spearman rank correlation tests. Statistical significance is reported at the 5% level.

All interviews were recorded digitally, transcribed, and analyzed using thematic analysis as advocated by Braun and Clark [26] using NVivo 11.4.1.1064. As with convergent mixed methods, researchers working on the project concurrently conducted the quantitative and qualitative elements, analyzed the two components independently, and interpreted the results together [27].

## Results

### Patient-Reported Outcome Measures (PROMs)

PROMs were available for 18, 15, and 9 patients at T1, T2, and T3, respectively.

The completion rate of the PROMs was over 90%: 97.2%, 100%, and 93.8% for SPARC at T1, T2, and T3, respectively; 100% for TAM and LCSS-Meso at all time points.

No significant changes in PROMs (Table 2) were observed, as measured on the LCSS-Meso, from T1 to T2 or T3 using the Wilcoxon matched pairs test (*P*=.293; Table 2). Even though this was a feasibility study and therefore not powered to detect statistical significance, we did note a statistically significant (*P*=.049) improvement from baseline to T2 (6-8 weeks) in psychological need on the SPARC scale, which suggests that completing a DSQ and using ASyMSmeso appear to provide psychological support to patients. Also, a statistically significant (*P*=.022) improvement was observed in the “Usefulness” domain of the TAM from T1 to T2, which suggests that patients found the ASyMSmeso system more useful over time. No statistically significant changes were identified at T3, but the low sample size at T3 renders analysis impractical.

**Table 2:** Patient-reported outcomes.

Measurement		T1^a^, mean (SD, range)	T1, median (IQR)	T1-T2^b^, median (IQR)	*P* value^c^	T1-T3^d^, median (IQR)	*P* value^e^
**SPARC^f^**							
	Physical	12.88 (8.87, 2.00-31.50)	12.53 (13.5)	2.00 (10)	.196	4.50 (7.25)	.123
	Psychological/emotional	3.28 (2.89, 0.00-9.00)	3.00 (5.25)	1.00 (2.00)	.049	1.00 (2.5)	.572
	Spiritual/religious	0.67 (0.97, 0.00-3.00)	0.00 (1.25)	0.00 (1.00)	.161	0.00 (1.00)	.102
	Independence	1.11 (1.23, 0.00-4.00)	1.00 (2.00)	0.00 (0.00)	1.00	0.00 (1.50)	.180
	Family/social	1.00 (0.89, 0.00-3.00)	1.00 (1.50)	0.00 (0.5)	1.00	0.00 (0.75)	1.00
	Treatment concerns	1.24 (1.56, 0.00-6.00)	1.00 (2.00)	0.00 (3.00)	.755	0.00 (2.5)	.683
**TAM^g^**							
	Ease of use	4.93 (1.08, 3.33-7.00)	5.00 (2.09)	-1.17 (1.67)	.196	–0.50 (2.00)	.352
	Usefulness	5.94 (0.84, 4.00-7.00)	6.00 (1.1)	0.00 (3.4)	.022	–0.30 (3.4)	.671
LCSS-Meso^h^		23.40 (18.12, 2.25-63.75)	13.69 (31.38)	–0.38 (26.88)	.859	–0.25 (28.06)	.575

^a^T1: baseline.

^b^T2: 6 weeks or 8 weeks.

^c^Comparing the change in median values from T1 to T2.

^d^T3: end of the study.

^e^Comparing the change in median values from T1 to T3.

^f^SPARC: Sheffield Profile for Assessment and Referral for Care.

^g^TAM: Technology Acceptance Model.

^h^LCSS-Meso: Lung Cancer Symptom Scale-Mesothelioma.

No statistically significant associations were found in the relationships between demographic or clinical variables and PROMs other than between age and the change from T1 to T2 in family/social issues on the SPARC questionnaire (*P*=.036), with a positive correlation coefficient of 0.584, indicating that for lower age groups, social and family issues increased over time.

### Adherence to the ASyMSmeso Intervention

Adherence to the ASyMSmeso intervention was explored. In total, 18 patients completed a total of 1343 DSQs over a total of 1334 days. There were more responses to DSQs than days because patients could complete them whenever they felt unwell or a symptom had changed. The compliance rate was 88.5%, with a median of 93.2% for completing DSQs. This rate of compliance is especially impressive given that the average number of days a person took part in the study was 82.8 days. This suggests that those taking part in the study found completing DSQs to be both feasible and acceptable.

### Alerts

Of the symptoms assessed on the DSQ, breathlessness was the most common symptom to generate an alert, followed by pain. There was a large number of additional symptoms that triggered alerts for individual patients such as “runny nose,” “stiffness in the hips,” and “urine infection.” The number and types of alerts that were generated by the system were analyzed; 7 patients did not generate any alerts during the study. The median number of alerts generated per patient was 3. Most patients generated an alert fewer than 10 times. One patient was responsible for a third of all alerts, with 52 alerts, and the 4 highest alerting patients were responsible for 116 of the 154 total alerts. This equates to one alert generated approximately every 8.33 days per patient. The highest alerting patient generated 1 alert per day during the study. The alerts occurred mostly in the early morning and early evening. As a result, there was a proportion of alerts that were out of hours (ie, after 5 pm), which warrants consideration for future implementation.

Health professionals had 24 hours to respond to the alerts generated by the system. Following an alert, patients were contacted mainly by telephone, and the most common response to the alerts was “advice as before,” primarily for the 1 patient who alerted daily with the same set of additional symptoms. Other responses centered on advice for supported self-care and how to access community or hospital services.

### User Experience

Participating patients, their carers, and clinicians were invited to take part in an end-of-study focus group or semistructured interview to explore their experiences and perceptions with using the ASyMSmeso system and identify how the implementation of ASyMSmeso impacted existing oncology services. The decision as to whether an interview or focus group was held depended on the availability of the participants.

#### Patient and Carer Experiences

A total of 8 patients and 3 carers were interviewed at the end of the study. All those recruited to the study were approached to take part in interviews. However, a number declined, and 1 person had died, giving a 47% response rate. Most participants were male (n=6), with an average age of 71 years. Two themes emerged from the interviews with patients and carers, namely (1) positive experiences and ease of use and (2) feelings of reassurance.

Regarding the theme of positive experiences and ease of use, in general, patients found the system very easy to use and quickly embedded it into their daily routine. They reported that it took them less than 5 minutes to complete the DSQ, and they usually completed it at the same time every day, often first thing in the morning. The system was described as “straightforward … and so simple to use” [Patient 01].

Very few issues with using the system were noted, and all participants felt that the DSQ was relevant to people with MPM:

…my activity level had changed; my walking ability had changed because of the breathlessness. So yeah, these were relevant to what was happening to me at that point in time.

Patient 03

Patient 03 felt that the symptom monitoring was relevant to what was happening to him in terms of his mobility and identified the link with his breathlessness.

Regarding the theme of feelings of reassurance, for many people, the sense of reassurance offered by the system was important, whether or not they were experiencing symptoms. One patient reported how “it was reassuring to know that you’re feeling stable and not having symptoms” [Patient 09]. Patient 09 felt reassured that there was no change in his symptom profile, and he felt stable; therefore, no recent sign of deterioration was reassuring to him.

For some, knowing that someone was listening was important:

I think what was good was the fact that you always felt that there was somebody else at the other end of the line who was listening to what you were saying.

Patient 01

This was an interesting finding that this patient felt he was being listened to: a sense of connectivity that brought a level of reassurance to him.

This feeling of connectivity was also mentioned by Patient 02 who felt reassured that someone was “watching him.”

Others spoke positively about responses to alerts, which resulted in their symptoms being effectively managed:

I kept getting bothered with my hips, and she said “have you taken anything for it?” She’d actually phoned me up, and I had said no. She said, what about paracetamol, and I said I’ll give it a go. So, I gave it a go, and it fixed it [laughs].

Patient 03

The patients reported little trouble in using the mobile phone to report symptoms; in fact, they found this experience reassuring, particularly when there was no change in symptoms over time and they could conclude that they were stable. The connectivity provided by the system was found to be reassuring to patients that someone was keeping an eye on them and responding to their data by calling them back and helping to sort out the problem.

#### Health Care Professionals’ Experience

At the end of the study, 11 health professionals with many years’ experience caring for patients with MPM (2 respiratory consultants, 4 clinical nurse specialists, 2 oncology nurses, 3 research nurses) took part in the interviews. From the interviews, 3 themes emerged: ease of use, symptom prevention and management, and enhanced communication.

Regarding the ease of use theme, similar to the feedback from the patients, the majority of health care professionals found the system acceptable and easy to use:

It was good. I found that really easy…

HP 11

Access to a smartphone device makes sense.

HP 09

They spoke about how, in general, they found the system easy to use and navigate. They also highlighted the benefit to both patients and themselves in achieving a better patient experience:

And, you know, badging this as a patient experience or patient experience improvement, and if there’s improvement in that, then that’s better for both the patient but also the treating clinicians.

HP 08

Some clinicians referred to the challenges of using remote monitoring concerning fears of technology and change:

You know, as always, it’s fear of change, isn’t it? Fear of technology, fear of change, fear of who is going to be responding to these alerts, and will it mean more work? So, I think that allaying those fears of change, getting buy-in from clinicians would probably overcome that.

HP 06

Some of the issues in the transition and ease of change to remote monitoring appeared unique to the health professionals, especially the fear of change, as expressed in the previous paragraphs. Interestingly, the patients did not identify any fears of using technology. The health professionals were fearful of the potential for increasing workload, handling and responding to alerts, and the need to work at allaying fears as part of the intervention.

Regarding the theme of improvement in symptom prevention, clinicians spoke about the positive clinical benefits of the alerting system — particularly its role in the early management of symptoms. One clinician described how signs of a chest infection were picked up, which then in turn led to “identification of disease recurrence” [HP 04].

Clinicians also spoke about how symptoms that may otherwise have been missed were picked up earlier:

Whereas maybe a patient wouldn’t necessarily call us for that, but we can call them back because you are noticing some changes in symptoms…

HP 04

One clinician found the system useful as a means of prioritizing care to those patients who were reporting more symptoms than others about things that they were not previously aware of:

It made me think about the way I assess people and what I ask them over the phone. Because sometimes what they alerted, [it] wasn’t something they told me about when I spoke to them or when I saw them in clinic. Like maybe they were reporting on that [DSQ] that they hadn’t eaten for a couple of days; then, when you see them in clinic, it’s like “oh yeah, I’ve got a diet. I’m eating really well. The wife’s making me a dinner.” So, it was... probing a wee bit better into what was actually going on.

HP 02

HP02 identified the benefits she experienced regarding her patient assessment and how the system was beneficial for probing patients further about their diet and nutrition, for example, and getting a better history of what was going on with the patient.

Another professional commented on how the data on the ASyMSmeso system regarding what symptom management interventions were used following an alert would give them a facility to look back and see what treatments were helpful for the patient in the past. This would prevent the future use of clinical management strategies that were already known to be of limited benefit to that person.

Regarding the theme of communication and connectivity, a few clinicians thought that those with MPM may not want to dwell on symptoms every day and would therefore not benefit from using the system — particularly if they had no symptoms at all:

I would think that, say “this just reminds me of the fact that I’ve got cancer. This doesn’t help me.”

HP 05

This finding is interesting in light of comments from patients, many of whom felt that even if they had no symptoms, filling in the DSQ and noting symptoms were stable resulted in feelings of positive wellbeing and reassurance.

Similar to the patients, clinicians also recognized the benefits of changing the mechanisms by which patients communicated with them — particularly the way that the mobile phone system did not rely on the patient contacting the professional directly but did this automatically — reporting issues in a timelier manner:

I think for the patients, it may mean that they don’t have to make that phone call. Because they just do it on the device and it goes through…I think ultimately that is generally a better thing, if they can let us know what is going on sooner rather than later.

HP 05

This anticipatory and preventative approach to care was also mentioned by HP03 as a good approach to having “infrastructure to manage it and monitor. And identify patients who are alerting and have early intervention to prevent deterioration in the symptom.” [HP03]

However, there was also some frustration that unchanging symptoms continued to alert. For example, if someone continued to report severe pain, the system would keep alerting until the symptom severity reduced to a lesser level. This led to some health professionals suggesting that the alerting algorithm needed adjusting and to be more refined at an individual level.

Regarding the flow of information available in the eLibrary, one health professional suggested that the eLibrary could be extended:

To cover things like travel insurance and all the other little things if they could use it almost as their own personal sort of reference thing.

HP 08

Some of the tips were good though, I think. That again, I’m not sure if the patients took on board that bit of it.

HP 10

Overall, the clinicians felt that the system was easy to use and enabled them to work together with the patient to improve their experience and manage symptoms in a timely manner. They also identified that the system enabled a preventative approach to symptom monitoring and could potentially enable early intervention. Unlike the patients, the clinicians identified some drawbacks to the system, such as leading to increased workload in managing alerts and feeling that patients might not want to be reminded of symptoms every day.

#### Suggested Improvements

While people with MPM and clinicians reported the ASyMSmeso system easy to use, there were suggestions to improve system function. These included having the option for an alarm to remind patients to complete the DSQ. The system as it is currently configured requires clinicians to log into a laptop or desktop computer to view patient symptom reports and to handle alerts (Figure 1). A number of health professionals suggested that it would make sense to put this functionality on the clinician’s mobile device. Other suggestions included amending the alerting algorithm to prevent over-alerting for unchanging symptoms.

## Discussion

### Principal Findings

The results demonstrate that this model of ASyMSmeso, a technology-enabled remote symptom monitoring system using PROMs, is feasible and acceptable to this population and those caring for them. Importantly, the findings provide new insights into effective ways to enhance the delivery of supportive care to people with MPM now and in the future, to improve patient outcomes and wellbeing. Our study demonstrated that those with MPM found the ASyMSmeso system easy to use, found it more useful over time, kept using it consistently for prolonged periods (2-3 months), and could see the value of using this remote technology in the management of their symptoms. These findings are notable, as it is well recognized that adherence, ease of use, and perceived usefulness are key factors in determining the successful implementation and scaling up of digital health technologies in health care contexts [24,28].

Supporting such positive findings is also the strong sense of reassurance that people with MPM expressed as a result of using this system. The virtual line of connectivity between patients and clinicians created by the ASyMSmeso system resulted in patients feeling a sense of reassurance and safety — knowing that there was “someone” at the other end watching over their symptoms and offering assistance if required. Such experiences resonate with other studies exploring the use of technology for the provision of supportive care to people with cancer [16,29]. A review of 14 qualitative studies exploring the use of telecare by people with cancer reported similar feelings of safety and reassurance and perceived improved communication with their health professionals as a result of using such systems [15]. The findings therefore are in agreement with previous studies conducted in other cancer populations that demonstrate that the monitoring of patient-reported outcomes using electronic platforms and devices is feasible and acceptable and has a positive impact on care outcomes [13,17,30]. In our study, we also found a complementary benefit identified by the clinicians in terms of ease of use and identification of a preventative approach to symptom management enabled by communication and connectivity in the system.

Furthermore, of note is the significant reduction in psychological needs observed from baseline to 6-8 weeks post-study. While the small number of participants recruited to the study and the focus on feasibility limit inferences made from this finding, it is possible that such reductions in psychological need may well be based on the positive perceptions and experiences of patients using the system. A trial evaluating electronic symptom reporting in people with metastatic cancer reported improvements in survival compared to standard care [13]. It may be well worth exploring whether such benefits related to survival transfer to people with MPM — particularly in light of the lack of curative treatments at the present time.

In terms of changes in practice, a number of clinicians spoke about how using the system made them change the way that they assessed symptoms and prioritized care. They recognized that, by remotely monitoring symptoms on a daily basis, they had a much more detailed picture of the individual’s symptom experiences over time, which could positively inform decision making and selection of supportive care interventions. Clinicians also identified benefits in changing the way that patients communicated with them — moving from a system that relied on patients recognizing symptoms and making a conscious decision to contact them to a system that reviewed patient symptom reports and automatically triggered alerts. Clinicians spoke about the benefits of the DSQ reporting of symptoms, particularly for those patients who were reluctant to bother clinicians by calling them to ask for help. These findings are of value in light of studies reporting that people with cancer continue to delay reporting life-threatening symptoms to health professionals [31].

The number of alerts generated throughout the study was relatively small and manageable. However, a significant number of alerts was out of hours. This warrants further investigation in future studies to understand why patients reported outside of normal working hours and what this means in terms of service provision. Furthermore, for the purpose of this study, clinicians managed alerts within the confines of their current practice (eg, 9 am to 5 pm, 7 days a week).

The PROMs used in the study were quick and easy to complete, with the LCSS-Meso taking about 4 minutes to complete and the SPARC, a holistic needs assessment tool, also quick and easy to complete. Adherence with completing these tools, including TAM, was very good, indicating the suitability for use in future trials.

### Limitations

Limitations of this study include that there was a decline in the PROM response rate across the study time points. This was due in part to research nurses not always being able to collect data at the agreed time points and in part as a result of the change from a 3-month to 2-month data collection protocol, which reduced data collection from 3 to 2 time points.

Participation rates were low for the study, and it is possible that some of those who declined to participate in the study were doing so because they did not want to think about their symptoms on a daily basis, a possibility also raised by some health professionals. However, only a small number of nonrespondents (n=2) explicitly gave this as a reason. It is also true that study participation rates can be very low among people with advanced cancer [32], and a number of personal, social, and systemic factors have been identified that have an impact on a patient’s decision to participate [33].

### Conclusion

In conclusion, the results of this study demonstrate, for the first time, that the remote monitoring and management of symptoms of people with MPM using ASyMSmeso are feasible and acceptable, and the evidence generated strongly supports future studies scaling up this digital intervention to a wider cohort of patients with MPM.

## Abbreviations

ASyMS: Advanced Symptom Management System
DSQ: daily symptom questionnaire
ECOG: Eastern Cooperative Oncology Group
eLibrary: electronic library
LCSS-Meso: Lung Cancer Symptom Scale-Mesothelioma
MPM: malignant pleural mesothelioma
PROM: patient-reported outcome measure
SPARC: Sheffield Profile for Assessment and Referral for Care
TAM: Technology Acceptance Model
